# Robust meal delivery service for the elderly: a case study in Hong Kong

**DOI:** 10.1038/s41598-022-25924-6

**Published:** 2022-12-13

**Authors:** Shaochong Lin, Monique Bakker, Eman Leung

**Affiliations:** 1 Department of Industrial and Manufacturing Systems Engineering, The University of Hong Kong, Pokfulam, Hong Kong; 2 Department of Management Sciences, City University of Hong Kong, Kowloon, Hong Kong; 3 School of Public Health and Primary Care, Chinese University of Hong Kong, Shatin, Hong Kong

**Keywords:** Health care, Engineering, Mathematics and computing

## Abstract

Service providers in a community center in Hong Kong deliver meals to community-dwelling elderly, first from a central kitchen to intermediate depots by a van and then to the homes of the elderly via walking. We propose a modified two-echelon vehicle routing model with concerns of both delivery efficiency and workload fairness among workers, incorporating important practical aspects, such as continuity of care and unique features of buildings and served elderly. Notably, we employ robust optimization to address service time uncertainties that differentiate between frail and ordinary elderly. The robust model can be transformed into a mixed integer program, for which we provide two decomposition-based approaches to accelerate computation. Through a real-data case study, we verify the effectiveness of the proposed models. We show that robust solutions can protect against service time variations and achieve better performance while incurring a small additional cost over deterministic ones. We provide insights into choosing the level of conservatism and human resource planning for practitioners.

## Introduction

Demand for home care services is growing rapidly in the face of aging populations. Most developed cities, including Hong Kong (HK), face the population aging issue. The proportion of elderly persons aged 65 and over in the total population in HK rose from 13% in 2011 to 20% in 2021^1^. The elderly are an especially vulnerable group to develop chronic diseases, and some chronic conditions would lead to mobility limitations, which are associated with increased fall risk, hospitalization, a decreased quality of life, and even mortality^2^. This is particularly true in light of the continuing COVID-19 pandemic, where the elderly are vulnerable with a high risk. To support activities of daily living and instrumental activities for the elderly, the healthcare and elderly care institutions have provided the elderly living in the community with home care services, such as meal delivery and housekeeping^3, 4^.

In HK, the Integrated Home Care Services Teams (IHCSTs) provide home-based community care and support to elderly persons aged 60 or above living in the community according to their individual needs. The services provided by IHCSTs aim at facilitating service users to continue aging in place for as long as possible and maintaining their optimal level of functioning while providing various kinds of support and assistance to carers^5^. The integrated home care services for the elderly are further classified into frail cases and ordinary cases, which are assessed under the Standardised Care Need Assessment Mechanism for Elderly Services^5^. The frail elderly are someone with disabilities, mobility limitations, or even on the wheel during daily life. Due to a severe shortage of frontline workers, a high turnover rate in staff, and a limited financial budget, the IHCSTs struggle to deliver effective home care services in time. This makes home care services much more demanding for the elderly in HK. According to the Social Welfare Department’s information, the average annual numbers of elderly persons on the waiting list under the ordinary cases and frail cases are about 4160 and 4624, respectively^6^. Among the frail cases, the annual increasing rate in the number of persons on the waiting list is about 28.6%, and the average waiting time is about 10–13 months to receive community-based care services^6^. Such kind of mismatch between the increasing demand for home care services and the limited supply of human and financial resources necessarily requires a highly effective and efficient delivery.

**Figure 1:**
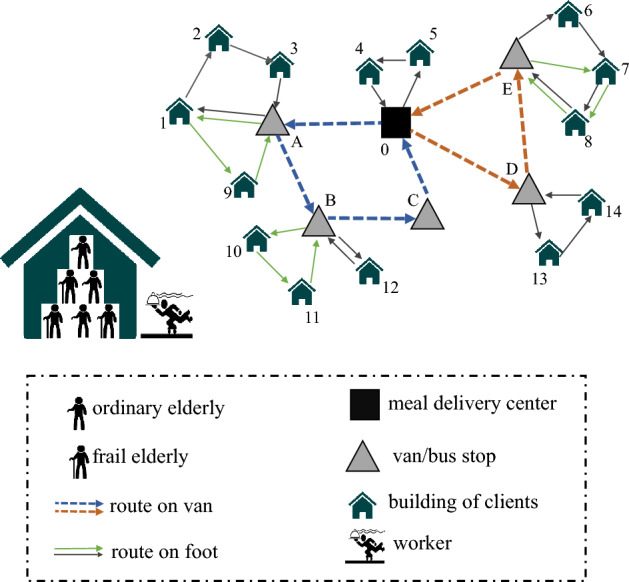
An example of two-echelon meal delivery in HK.

An IHCST is a pool of experienced and professionally trained staff. There are a total of 61 IHCSTs in HK, and each IHCST has its community-based service boundary of buildings where the served clients (i.e., the elderly in service demand) live. Each IHCST should assign daily delivery tasks and plan delivery routing (i.e., sequences of served clients) for workers. Although IHCSTs have their delivery vehicles (vans), most of the deliveries cannot directly go from distribution centers to the clients via van due to the unique features of the clients’ buildings in HK. The space between neighboring buildings in HK is always very narrow. The public housing estates are high-rise, and several clients may live in the same building. In practice, home care services delivered by an IHCST from a center to clients are made by two types of transportation, i.e., first by van and then on foot. In this work, we model such kind of service deliveries as a modified two-echelon vehicle routing problem (2E-VRP). In classic 2E-VRPs in the literature, the freight delivery from the depot to the customers is managed by shipping the freight through intermediate depots^7^. In terms of the home care services in HK, the transportation network is decomposed into two levels, where the 1st level connects a center to the intermediate depots (say van stops or other types of drop-off locations) and the 2nd connects the intermediate depots to the buildings of clients. Without loss of generality, we will use meal delivery as a case to illustrate home care services delivered by IHCSTs in the remaining context, as shown in Fig. 1.

To assign delivery tasks and plan delivery routing for workers on a complex two-echelon network is challenging (as shown in Fig. 1). The current practice of IHCSTs still relies on a manual approach to generate task assignments and routing solutions, which is experience-based, with resulting solutions always far from optimal. It is highly demanding to propose a scientific approach to it. Based on consultations with a center manager, we note that working times spent on deliveries variate among workers mainly due to uncertain service times, which has been one of the main reasons for high turnover rates because of uneven and over workload, causing the feeling of unfairness among workers. In addition, as the workers may share the same van to return to the delivery center after finishing the assigned service tasks, ensuring a fair workload is also for efficiency. Thus, both center managers and workers find fairness to be very important. In this study, we use a min-max measure over working times spent by different workers (i.e., to minimize the “makespan”). According to historical observations in practice, service times largely variate case by case, and the fluctuations differentiate between ordinary cases and frail cases. A frail elderly, as opposed to an ordinary elderly, typically requires more time when receiving the same service, and service time for them is more likely to show larger variations. For the case of meal delivery, uncertain service time may lead to scenarios in which the realized workloads of some workers are extremely long, and the time of some clients to receive the meal has been out of the proper lunch or dinner time window. The above-mentioned challenges have been a huge burden on already cash-strapped IHCSTs. In this study, our objective is to provide a scientific approach for robust route design and task assignments against uncertain service times.

## Related work

The existing studies on home-care operations focus on vehicle routing and scheduling to perform various services at clients’ homes/sites using operations-research modeling (see reviews^3, 4^). Utilizing mathematical modeling and computing methods has shown an increased operational efficiency by 10–15%^8^, and savings ranging from 5 to 20% of the total costs^9^, compared with manual approaches. The home care service problems in the literature are formulated as a classic vehicle routing problem (VRP)^3, 10^. Differently, due to the mentioned unique features of buildings in HK, we model the meal delivery service problem as a modified 2E-VRP. The two-echelon version of the VRP (i.e., 2E-VRP) was considered in freight distribution systems for congested urban areas^11, 12^ and was first formally studied in 2011^7^, where the delivery from the depot (i.e., delivery center) to the customers is managed by rerouting and consolidating the freight through different intermediate satellites. In our meal-delivery problem where van stops correspond to the intermediate satellites (as shown in Fig. 1), the focal setting has some salient differences compared with classic 2E-VRPs. First, the demand at the 2nd-level served points in classic 2E-VRPs cannot be split among different service providers^7^. On the contrary, the same building of clients can be served by more than one worker in our setting, e.g., buildings 1, 7, and 8 in Fig. 1. Thus, the aggregated demand in a building should be split into client-based points in our model. Second, in our meal-delivery case, the delivery is allowed to go directly from the delivery center to the clients, e.g., the routing of 0-5-4 in Fig. 1. For classic 2E-VRPs, instead, the freight must be consolidated from the depot to a satellite and then delivered from the satellite to the end customers. Third, the existing studies on classic 2E-VRPs consider deterministic settings or depend on a two-stage stochastic setting for tactical planning^13^. We focus on 2E-VRPs under uncertainty, which has been largely ignored.

To address the service time uncertainty and protect against the extremely long working time for some workers, we utilize robust optimization techniques in our modified 2E-VRP incorporating uncertainty. Robust optimization has been widely applied to cases with partial knowledge of the underlying distribution^14^. Various studies have developed effective reformulations and algorithms for generic optimization problems with uncertain parameter sets^15–18^, with applications in many different areas including transportation^4^, engineering^19^, and healthcare setting^20^. In our meal-delivery case, we specify two uncertain-but-bounded perturbation sets separately for service times of the ordinary and frail elderly, where parameters of the uncertainty set are obtained based on historical observations and consultation. For the frail elderly, due to the different magnitudes of mobility limitations (e.g., meal-on-wheel^21^), the service time is relatively long with a greater perturbation range compared with the ordinary ones. We employ a budget-constrained robust model^22^, which is featured with a budget on uncertainty to control the level of conservatism (i.e., to control the percentage of served clients’ service time that can take their extreme duration values). The resulting solution, subject to the budget constraint, will become safe and perform well even under highly uncertain scenarios. In this study, we separately specify the budgets on the service time uncertainty for the ordinary and frail elderly.

Our proposed robust optimization model can be applied to routine community-based home care services in densely populated and aging cities (e.g., HK). The contributions of this work are summarized below.

- Due to the unique features of residential estates in HK, we first model the home care services as a modified two-echelon vehicle routing problem (2E-VRP), incorporating concerns of both efficiency and fairness under a meal-delivery case. As to be introduced in the next section, our model also incorporates other important practical aspects, such as “continuity of care” and features of buildings (e.g., setup time, worker capacity, with or without a lift).
- We propose a robust 2E-VRP model under service time uncertainty that is differentiated between frail and ordinary elderly. We show that the robust model can be transformed into a mixed integer program (MIP), for which we provide two decomposition-based approaches to ensure computation efficiency.
- Through a real-data meal-delivery case study, we verify the effectiveness of our modified 2E-VRP model and its robust version, with improving on average 4.7–7.0% delivery efficiency compared with the operations implemented by IHCSTs. The robust solutions can protect against service time variations and achieve better performance while incurring a small additional cost over deterministic optimal solutions. We also provide managerial insights regarding risk management and long-term human resource planning.

The remainder of the paper is organized as follows. First, the problem is described, and formulations (deterministic and robust counterparts) are presented. Second, two decomposition-based algorithms are developed for the computation of the resulting MIP. Third, the numerical results of the case study and discussions are presented. Last, conclusions and directions for future work are discussed.

## Model formulations

In this section, the model development for meal-delivery planning is presented. First, we define our nominal (deterministic) model, followed by its robust counterpart and its linear equivalent.

### Problem description

We use a network $G=(N, A)$ to describe our problem where *N* is a node set and *A* is an arc set. We define *K* as the set of meal-delivery workers and *B* as the set of buildings that need to be served. In our meal-delivery network, $N = \{0, e^* \} \cup B \cup S$, i.e., the node set is comprised of the delivery center (node 0), the dummy ending point $e^*$, a set of served buildings *B*, and a set of van stops *S* that can be traversed. Van stops are intermediate parking areas or stations pre-specified for delivery vans, similar to bus stops. Let *L* be the full client set and $L_i$ be the set of clients to be served at building $i\in B$. The number of clients to be served in building $i\in B$ is $|L_i|$. Before delivery, the information about clients from all different buildings is known, and the demanding services must be fulfilled. All workers (i.e., $\forall k\in K$) start at the same time from the same delivery center (node 0). As mentioned, clients’ buildings are reached through a 2-echelon delivery: first via van and then on foot. We have the arc set $A=\{(i,j): i, j\in N, i\not = j\}$, where the related distance for each arc is expressed in travel time $\tau _{ij}$. We refer $A_S$ ($A_S\subset A$) to the set of arcs linking van stops and the delivery center. Specifically, for $(i, j)\in A_S$, we refer $\tau _{ij}$ to the travel time by van, and for arcs not belonging to $A_S$, we refer $\tau _{ij}$ to the travel time on foot. The route via van is comprised of some linkages among |*S*| van stops in sequence. For each delivery (i.e., lunch or dinner), each worker is assumed to get off at no more than one van stop. In other words, once a worker gets off the van at a certain stop, he/she cannot go back onto the van before he/she finishes the assigned delivery jobs, i.e., visiting and serving his/her assigned clients on foot sequentially. Note that workers can also directly go from the delivery center to reach clients on foot. This permission largely expands the solution space of the 2-echelon setting, bringing challenges in computation. After finishing all of the assigned jobs, the workers will go back to the delivery center. The time spent on the back journey (i.e., $\tau _{ie^*}$) is calculated by the shortest time spent from node *i* to the delivery center (on foot and by van). As customers may share the same van and thus need to wait for the ones who are relatively late to arrive at van stops, without further justification, we use a dummy point $e^*$ instead for simplicity.

The objective of task assignments and route design is to maximize delivery efficiency while ensuring the fairness of working time among workers. The total working time for each worker includes the time spent on the road, entering a building, and serving frail and ordinary elderly. The delivery center promises the clients to obtain their meal at a regular 1-hour time interval (e.g., 11:30 - 12:30 for lunch and 17:30–18:30 for dinner). Although the objective of the current study is worker-orient, improving the delivery efficiency is also to ensure that clients can receive the meals on time. Workload fairness among workers is important in many planning, scheduling, and resource allocation problems^23^. When route design and task assignments are done through mathematical optimization rather than by hand, perceived political issues can be avoided, since workers no longer doubt the fairness of the method used to allocate workload^24^. A min-max fairness approach can balance the workload among workers in home-care service problems^25^. In our model, we adopt such a min-max measure to balance the fairness of workload among workers (to be introduced in formula (1)). Besides, we consider continuity of care, i.e., “having the same care provider who knows and follows the patient$''$, which is important for home-care service^26–29^ because it fosters improved communication, trust, and a sustained sense of responsibility^30^. Continuity of care can also be reflected in a caregiver-client relationship where there is a preference for each caregiver serving the same, or similar, set of clients^27^. In our model, we assume that each worker has a prespecified set of his/her familiar clients, and additional penalty time is incurred when a worker serves a client he/she is unfamiliar with. In the context of meal delivery, no penalty reflects the worker’s ability to quickly and easily find his/her way to a client’s living place. With such a consideration, the worker becomes “heterogeneous” in terms of the delivery time for familiar or unfamiliar clients, which brings new challenges in modeling and computation.

The set notations, parameters, and decision variables used throughout the paper are summarized in Tables 1, 2 and 3. We first have a deterministic model (DM) where the service time is assumed to be certain.

**Table 1:** Definitions for sets.

Notation	Meaning
$L^f$	set of frail clients $l= 1, \ldots , |L^f|$
$L^o$	set of ordinary clients $l= 1, \ldots , |L^o|$
*L*	union of sets $L^f$ and $L^o$ including all of the clients, i.e., $L = L^f \cup L^o$
*B*	set of all served buildings $i=1,\ldots , |B|$
$L_i$	set of clients living in building $i (i\in B)$
*K*	set of workers $k=1, \ldots , |K|$
*S*	set of van stops $i=1, \ldots , |S|$
*N*	set of all nodes, $N= S\cup B \cup \{0, e^*\}$
*A*	set of all arcs, $A=\{(i,j): i, j\in N, i\not = j\}$
$A_S$	set of arcs for vans, $A=\{(i,j): i, j\in S\cup \{0, e^*\}, i\not = j\}$

**Table 2:** Definitions for parameters.

Notation	Meaning
$m_v$	number of delivery vans
$m_s$	number of seats on each delivery van
*C*	total capacity of a worker
$C_i^*$	the capacity of a worker entering building *i* without a lift
$\alpha$	demand splitting parameter, $0.0\le \alpha \le 1.0$
$\tau _{ij}$	travel time from node *i* to node *j*
$\mu _l^k$	service time duration for client *l* served by worker *k*
$\upsilon _i$	setup time duration to enter into building $i (i\in B)$, incurred once per building is served
$F^k$	the set of familiar clients for worker $k (k\in K)$
$\rho ^k_{l}$	additional penalty time taken by worker *k* to reach client *l*, incurred once $l\notin F^k$

**Table 3:** Definitions for decision variables.

Notation	Meaning
$y^k_{l}$	binary decision variable: 1 if worker *k* serves client *l*, 0 otherwise
$x^k_{ij}$	binary decision variable: 1 if worker *k* uses arc $(i,j)\in A$, 0 otherwise
$\phi _{ij}$	binary decision variable: 1 if arc (*i*, *j*) is used in route of some van, 0 otherwise
$z^k_{i}$	binary variable: 1 if worker *k* visits building *i*, 0 otherwise
$u_{i}^k$	dummy variable for sub-tour elimination
$T_k$	total time spent by worker *k*

1 $$\begin{aligned} {\textbf {(DM) }} \min \max _k&T_k = \sum _{(i,j)\in A} \tau _{ij}x^k_{ij} + \sum _{i \in B} \upsilon _i z^k_{i} + \sum _{l \in L}(\rho ^k_{l}+ \mu _l^k) y^k_{l} \end{aligned}$$

2 $$\begin{aligned} \text {s.t. }&\sum _{i \in N}x^k_{0i} = 1, \quad \forall k \in K, \end{aligned}$$

3 $$\begin{aligned}&\sum _{i \in N}x^k_{ij} - \sum _{i \in N}x^k_{ji} = 0, \quad \forall j \in N/\{0,e^*\}, k \in K, \end{aligned}$$

4 $$\begin{aligned}&\sum _{i \in N}x^k_{ie^*} = 1, \quad \forall k \in K, \end{aligned}$$

5 $$\begin{aligned}&\sum _{i \in N}x^k_{ij} - z^k_{j} = 0, \quad \forall j \in B, k\in K, \end{aligned}$$

6 $$\begin{aligned}&x^k_{ij}- m_v\phi _{ij}\le 0, \quad \forall (i, j)\in A_S, k \in K, \end{aligned}$$

7 $$\begin{aligned}&\sum _{i \in N}\phi _{0i}\le m_v, \end{aligned}$$

8 $$\begin{aligned}&\sum _{i \in S}\phi _{ij} - \sum _{i \in S}\phi _{ji} = 0, \quad \forall j \in S, \end{aligned}$$

9 $$\begin{aligned}&\sum _{i \in S}\phi _{ie^*} \le m_v, \end{aligned}$$

10 $$\begin{aligned}&\sum _{k\in K}x^k_{ij}\le m_s, \quad \forall (i, j)\in A_S, \end{aligned}$$

11 $$\begin{aligned}&\sum _{k \in K}y^k_{l} = 1, \quad \forall l\in L, \end{aligned}$$

12 $$\begin{aligned}&|L_i| z^k_{i}-\sum _{l \in L_i}y^k_{l} +1 \le |L_i|, \quad \forall i \in B , k \in K, \end{aligned}$$

13 $$\begin{aligned}&\sum _{l \in L}y^k_{l} \le C, \quad \forall k \in K, \end{aligned}$$

14 $$\begin{aligned}&\sum _{l \in L_i}y^k_{l} \le C_i^*, \quad \forall i\in B, k \in K. \end{aligned}$$

15 $$\begin{aligned}&\sum _{k \in K}z^k_{i} \le \lceil |L_i|/(\alpha C)\rceil , \quad \forall i \in B, \end{aligned}$$

16 $$\begin{aligned}&u_{i}^k-u_{j}^k + (|N|)x^k_{ij} \le |N|-1, \quad \forall (i, j) \in A, k \in K, \end{aligned}$$

17 $$\begin{aligned}&x^k_{ij} \in \{0,1\}, \quad \forall (i, j) \in A, k \in K, \end{aligned}$$

18 $$\begin{aligned}&z^k_{i} \in \{0,1\}, \quad \forall i \in B, k \in K, \end{aligned}$$

19 $$\begin{aligned}&y^k_{l} \in \{0,1\}, \quad \forall l \in L, k \in K, \end{aligned}$$

20 $$\begin{aligned}&\phi _{ij} \in \{0, 1\}, \quad \forall (i, j)\in A_S. \end{aligned}$$

21 $$\begin{aligned}&u_i^k \ge 0, \quad \forall i\in N, k \in K. \end{aligned}$$

The objective (1) is to minimize the maximum working time spent among workers. The total working time for each worker *k* includes the time duration on the road (i.e., time spent on van and on foot), setup time to enter buildings, and service time for the frail elderly and ordinary elderly. Constraints (2) and (4) ensure that every worker starts at the delivery center (i.e., node 0) and finishes at the dummy node. Constraints (3) and (5) take care of flow conservation for each worker *k* in nodes and arcs, respectively. Constraint (6) ensures that one worker should take on one van to traverse between van stops. Constraints (7)–(9) ensure the network flow balance of vans. Constraint (10) is the capacity for each van. Constraint (11) ensures that all demand is fulfilled, and constraint (12) ensures that visiting a building without filling any demand is not allowed. Constraint (13)–(15) are the capacity constraints on the total demand filled by each worker, demand filled by workers serving a building without a lift, and the number of workers to enter into the same building, respectively. Constraint (15) is called demand splitting where $\alpha$ is the parameter to control the magnitude of splitting, i.e., at most $\lceil |L_i|/(\alpha C)\rceil$ workers can serve building *i*. This splitting constraint is just following the practice. Constraint (16) is for sub-tour elimination. As described above, the DM model balances the delivery efficiency and fairness of workloads among workers, incorporating some important practical aspects, including building-specific features (e.g., setup time to enter into a building, workload capacity without a lift, demand splitting) and client-specific features (e.g., “continuity of care”, frail and ordinary cases). The DM model can also be reformulated as the following MIP formulation.

22 $$\begin{aligned}&\min \; T \end{aligned}$$

23 $$\begin{aligned} \text {s.t. }&\sum _{(i,j)\in A} \tau _{ij}x^k_{ij} + \sum _{i \in B} \upsilon _i z^k_{i} + \sum _{l \in L}(\rho ^k_{l}+ \mu _l^k) y^k_{l} \le T, \quad \forall k \in K, \end{aligned}$$

24 $$\begin{aligned}&(2){-}(21). \end{aligned}$$

### Model formulation under uncertainty

As introduced, the service times for clients are highly unpredictable with great variations, leading to uneven realized workloads among workers and further exacerbating delivery efficiency and staff shortage due to the high turnover rate. We model the uncertainty in service times for the ordinary and frail elderly (i.e., $\mu _l^o$ and $\mu _l^f$) separately as serving a frail elderly takes a relatively long time with a greater fluctuation range observed from historical practice. We employ box perturbation sets to model the service time uncertainty with $\mu ^k_{l}\in [{\overline{\mu }}^k_{l}-{\hat{\mu }}^k_{l},{\overline{\mu }}^k_{l}+{\hat{\mu }}^k_{l}]$, where ${\overline{\mu }}^k_{l}$ is the nominal value and ${\hat{\mu }}^k_{l}$ is the positive radius of perturbation set^18^. Specifically, $\mu _l^k$ is assumed from an unknown distribution, taking value in $[ {\overline{\mu }}_o - {\hat{\mu }}_o, {\overline{\mu }}_o + {\hat{\mu }}_o ]$ for the ordinary elderly and in $[ {\overline{\mu }}_f - {\hat{\mu }}_f , {\overline{\mu }}_f + {\hat{\mu }}_f]$ for the frail elderly, with ${\overline{\mu }}_f> {\overline{\mu }}_o$ and ${\hat{\mu }}_f> {\hat{\mu }}_o$. A most conservative way to protect against the worst-case scenario is to utilize robust optimization with the min-max format: $\min \max _{\mu _l^k} \{T, \text {s.t.} (23){-}(24)\}$, where the constraint (23) associated with uncertainty taking extreme values becomes

25 $$\begin{aligned} \sum _{(i,j)\in A} \tau _{ij}x^k_{ij} +\sum _{i \in B} \upsilon _i z^k_{i} + \sum _{l \in L^o}(\rho ^k_{l}+ {\overline{\mu }}_o + {\widehat{\mu }}_o) y^k_{l} + \sum _{l \in L^f}(\rho ^k_{l}+ {\overline{\mu }}_f + {\widehat{\mu }}_f) y^k_{l} \le T, \quad \forall k \in K. \end{aligned}$$

To control the level of conservatism, we further employ a budget-constrained approach that retains the advantages of computationally demanding linear programming^18^. We use parameters $\Gamma _o^k$ and $\Gamma _f^k$ to control the total deviation of the uncertain parameters (i.e., the budget of uncertainty) for the ordinary and the frail elderly, respectively. The goal of the budget-constrained approach is to protect against all cases in which up to $\lfloor \Gamma _o^k \rfloor$ coefficients (i.e., $\mu _l^k$) of ordinary service jobs are allowed to change by ${\hat{\mu }}_o$ and one coefficient changes by at most $(\Gamma _o^k - \lfloor \Gamma _o^k \rfloor ){\hat{\mu }}_o$, and $\Gamma _f^k$ is likewise for the frail service jobs. We then have the following robust optimization model (RO):

26 $$\begin{aligned} {\textbf {(RO) }}&\min \; T \end{aligned}$$

27 $$\begin{aligned} \text {s.t. }&\sum _{(i,j)\in A} \tau _{ij}x^k_{ij} + \sum _{i \in B} \upsilon _i z^k_{i} + \sum _{l \in L^o}(\rho ^k_{l}+ {\overline{\mu }}_o) y^k_{l} + \sum _{l \in L^f}(\rho ^k_{l}+ {\overline{\mu }}_f) y^k_{l} + \mathbf {\beta }^k_o+\mathbf {\beta }^k_f \le T, \quad \forall k \in K, \end{aligned}$$

28 $$\begin{aligned}&\mathbf {\beta }_o^k=\underset{ \{S_k \cup {t_k}|S_k \subseteq L^o, |S_k| = \lfloor \Gamma ^k_o\rfloor , t_k \in L^o\backslash S_k \}}{\max }\left\{\sum _{l\in S_k} {\widehat{\mu }}_o y^k_{l}+(\Gamma ^k_o - \lfloor \Gamma _o^k\rfloor ){\hat{\mu }}_oy^k_{t_k}\right\}, \end{aligned}$$

29 $$\begin{aligned}&\mathbf {\beta }_f^k=\underset{ \{S_k \cup {t_k}|S_k \subseteq L^f, |S_k| = \lfloor \Gamma ^k_f\rfloor , t_k \in L^f\backslash S_k \}}{\max }\left\{\sum _{l\in S_k} {\widehat{\mu }}_f y^k_{l}+(\Gamma ^k_f - \lfloor \Gamma _f^k\rfloor ){\hat{\mu }}_fy^k_{t_k}\right\}, \end{aligned}$$

30 $$\begin{aligned}&(2){-}(21). \end{aligned}$$

The budget constraints (28) and (29) in the RO are to model the magnitude of conservatism against uncertainty, with $\beta _o^k$ for the ordinary case and $\beta _f^k$ for the frail case, respectively. Each of them involves an inner optimization problem. Take the inner optimization problem in (28) as an example, $S_k$ is a decision variable set ($S_k \subset L^o$) corresponding to selecting up to $\lfloor \Gamma _o^k \rfloor$ coefficients and $t_k$ is the decision variable to select one coefficient that changes by at most $(\Gamma _o^k - \lfloor \Gamma _o^k \rfloor ){\hat{\mu }}_o$. Next, we show that the RO formulation has an equivalent MIP. Given ${\textbf{y}}$, problem (28) can be transformed^22^ as

$$\begin{aligned} \mathbf {\beta }_o^k= \max _{0\le q^k_l \le 1, \quad \forall l\in L^o}\left\{ \sum _{l\in L^o} {\widehat{\mu }}_o y^k_lq^k_l: \sum _{l\in L^o}q^k_l \le \Gamma _o^k\right\} , \end{aligned}$$

which is a linear program and the associated dual is:

31 $$\begin{aligned} \min&\sum _{l\in L^o} w_l^k+ \Gamma ^k_o r^k_o \end{aligned}$$

32 $$\begin{aligned} \text {s.t. }&r_o^k+w_l^k\ge {\widehat{\mu }}_o y^k_l, \quad \forall l\in L^o, \end{aligned}$$

33 $$\begin{aligned}&w_l^k \ge 0, \quad \forall l\in L^o, \end{aligned}$$

34 $$\begin{aligned}&r_o^k \ge 0, \end{aligned}$$

where $w_l^k$ and $r^k_o$ are the dual variables. By strong dual theorem, objective values of the original problem and dual problem coincide. In the same way, we have $\mathbf {\beta }_f^k=\min _{r_f^k \ge 0; w_l^k \ge 0, \forall l\in L^f }\{w_l^k+ \Gamma ^k_f r^k_f: r_f^k+w_l^k\ge {\widehat{\mu }}_f y^k_l, \forall l\in L^f \}$ and its dual format. Therefore, we have the following equivalent MIP formulation of the RO model:

35 $$\begin{aligned} {\textbf {(MIP) }}&\min \; T \end{aligned}$$

36 $$\begin{aligned} \text {s.t. }&\sum _{(i,j)\in A} \tau _{ij}x^k_{ij} + \sum _{i \in B} \upsilon _i z^k_{i} + \sum _{l \in L^o}(\rho ^k_{l}+ {\overline{\mu }}_o) y^k_{l} + \sum _{l \in L^f}(\rho ^k_{l}+ {\overline{\mu }}_f) y^k_{l} + \sum _{l\in L} w_l^k+ \Gamma ^k_o r_o^k+\Gamma ^k_f r_f^k \le T, \quad \forall k \in K, \end{aligned}$$

37 $$\begin{aligned}&r_o^k+w_l^k\ge {\widehat{\mu }}_o y^k_l, \quad \forall l\in L^o, k\in K, \end{aligned}$$

38 $$\begin{aligned}&r_f^k+w_l^k\ge {\widehat{\mu }}_f y^k_l,\quad \forall l\in L^f, k\in K, \end{aligned}$$

39 $$\begin{aligned}&w_l^k \ge 0, \quad \forall l\in L, k\in K, \end{aligned}$$

40 $$\begin{aligned}&r_o^k, r_f^k \ge 0, \quad \forall k\in K, \end{aligned}$$

41 $$\begin{aligned}&(2){-}(21). \end{aligned}$$

The MIP formulation (35)–(41) can be directly solved by some commercial optimizer (e.g., CPLEX or Gurobi). For large-scale instances, it is even time-consuming to find a feasible solution.

### Two decomposition-based approaches

The existing studies on algorithms for the deterministic 2E-VRP either consider metaheuristics such as adaptive large neighborhood search algorithm^31^ or column generation-based algorithms^32^. These approaches cannot be directly applied to our setting as we consider many important and practical features in our modified 2E-VRP model under uncertainty. We propose two decomposition-based approaches to accelerate the computation of the MIP.

First, we consider an easy-to-implement decomposition-based heuristic (DH) to reduce the complexity of the problem. We first decompose the problem into two segments: buildings directly served by workers on foot and buildings served by vans then on foot, according to the physical distances from the delivery center to the buildings. Such a segmentation strategy has been shown to be effective in practice^33^. After decomposing the service region into two segments (say on-foot segment and by-van segment), one needs to further assign workers to two segments. In our heuristic, we first determine the number of assigned workers according to the total worker-client percentage and then determine the on-foot workers based on the familiar set of workers. The worker with more familiar clients living in the on-foot segment is more likely to be selected as an on-foot worker. This strategy can largely reduce the solution space, making the resulting two subproblems solvable within adequate time. such a segmentation arises naturally since there is already a distinction between buildings served on foot and by van in practice. This practice-driven decomposition heuristic can also be extended to solving highly large-scale instances by decomposing the problem into more segments.

Second, through utilizing the structure of the MIP formulation, we consider a Benders decomposition (BP) approach^34, 35^ as the BP technique can well separate discrete variables of routing and assignment decisions and continuous variables from the transformed RO model. We briefly describe the BP framework for our meal-delivery context. In the MIP, given routing variables ${\bar{\textbf{x}}}$ and assignment variables ${\bar{\textbf{y}}}$ and ${\bar{\textbf{z}}}$, the resulting subproblem becomes the following linear programming:

42 $$\begin{aligned} {\textbf {(SP) }}&\min \; T \end{aligned}$$

43 $$\begin{aligned} \text {s.t. }&g({\bar{\textbf{x}}},{\bar{\textbf{y}}}, {\bar{\textbf{z}}} )+ \sum _{l\in L} w_l^k+ \Gamma ^k_o r_o^k+\Gamma ^k_f r_f^k \le T, \quad \forall k \in K, \end{aligned}$$

44 $$\begin{aligned}&r_o^k+w_l^k\ge {\widehat{\mu }}_o {\bar{y}}^k_l, \quad \forall l\in L^o,\quad \forall k\in K,\end{aligned}$$

45 $$\begin{aligned}&r_f^k+w_l^k\ge {\widehat{\mu }}_f {\bar{y}}^k_l, \quad \forall l\in L^f, \quad \forall k\in K, \end{aligned}$$

46 $$\begin{aligned}&(39){-}(41), \end{aligned}$$

where $g({\bar{\textbf{x}}},{\bar{\textbf{y}}}, {\bar{\textbf{z}}} )= \sum _{(i,j)\in A} \tau _{ij}{\bar{x}}^k_{ij} + \sum _{i \in B} \upsilon _i {\bar{z}}^k_{i} +\sum _{l \in L^o}(\rho ^k_{l}+ {\overline{\mu }}_o) {\bar{y}}^k_{l} + \sum _{l \in L^f}(\rho ^k_{l}+ {\overline{\mu }}_f) {\bar{y}}^k_{l}$. Let $\varvec{\xi }$ and $\varvec{\zeta }$ be the dual variable associated with constraints (43)–(45). The dual of the SP formulation is given by:

47 $$\begin{aligned} {\textbf {(DSP)}}&\max \sum _{k \in K} g({\bar{\textbf{x}}}, {\bar{\textbf{y}}}, {\bar{\textbf{z}}}) \xi ^k + \sum _{k \in K}\sum _{l\in L^o} {\widehat{\mu }}_o {\bar{y}}^k_l \zeta _l^k + \sum _{k \in K}\sum _{l\in L^f} {\widehat{\mu }}_f {\bar{y}}^k_l \zeta _l^k\end{aligned}$$

48 $$\begin{aligned} \text {s.t. }&\sum _{k \in K} \xi ^k \le 1, \end{aligned}$$

49 $$\begin{aligned}&\zeta _l^k - \xi ^k \le 0, \quad \forall l\in L, k\in K, \end{aligned}$$

50 $$\begin{aligned}&\sum _{l\in L^o} \zeta _l^k -\Gamma _o^k \xi ^k \le 0, \quad \forall k\in K, \end{aligned}$$

51 $$\begin{aligned}&\sum _{l\in L^f} \zeta _l^k - \Gamma _f^k \xi ^k \le 0, \quad \forall k\in K, \end{aligned}$$

52 $$\begin{aligned}&\zeta _l^k \ge 0, \quad \forall l\in L, k\in K, \end{aligned}$$

53 $$\begin{aligned}&\xi ^k \ge 0, \quad \forall k \in K. \end{aligned}$$

By strong duality, the optimal objective value of the primal problem (i.e., SP) is equal to that of its dual problem (i.e., DSP). Combining the resulting solution $({\bar{\textbf{w}}^*},{\bar{\textbf{r}}^*}, {\bar{T}}^*)$ with $({\bar{\textbf{x}}},{\bar{\textbf{y}}}, {\bar{\textbf{z}}})$, we obtain a feasible solution, providing an upper bound of the optimal objective value for the MIP. Note that the feasible region defined by (48)–(53) does not depend on the value of $({\textbf{x}}, {\textbf{y}}, {\textbf{z}})$. It is also straightforward to check that the optimal values of the SP and DSP are finite subject to non-empty region. Let $\varvec{\xi }^j (j\in {\mathscr{J}})$ denote all extreme points of the feasible region in the DSP, with $[\varvec{\xi }^j ]^k$ and $[\varvec{\zeta }^j ]^k_l$ be the associated values. The DSP then can be reformulated as:

54 $$\begin{aligned} \min \left\{ T: T\ge \sum _{k \in K} g({\bar{\textbf{x}}}, {\bar{\textbf{y}}}, {\bar{\textbf{z}}}) [\varvec{\xi }^j ]^k+ \sum _{k \in K}\sum _{l\in L^o} {\widehat{\mu }}_o {\bar{y}}^k_l [\varvec{\zeta }^j ]^k_l + \sum _{k \in K}\sum _{l\in L^f} {\widehat{\mu }}_f {\bar{y}}^k_l [\varvec{\zeta }^j ]^k_l, \quad \forall j\in {\mathscr{J}} \right\} . \end{aligned}$$

Therefore, the MIP can be reformulated as a master problem (MP) of the BP approach:

55 $$\begin{aligned} {\textbf {(MP)}}&\min \; T \end{aligned}$$

56 $$\begin{aligned} \text {s.t. }&T\ge \sum _{k \in K}[ \varvec{\xi }^j ]^k g({\textbf{x}}, {\textbf{y}}, {\textbf{z}}) + \sum _{k \in K}\sum _{l\in L^o} {\widehat{\mu }}_o [\varvec{\zeta }^j ]^k_l y^k_l + \sum _{k \in K}\sum _{l\in L^f} {\widehat{\mu }}_f [\varvec{\zeta }^j ]^k_l y^k_l, \quad \forall j\in {\mathscr{J}}, \end{aligned}$$

57 $$\begin{aligned}&(2)-(21), \end{aligned}$$

where constraint (56) is also called Benders cuts. It may be impractical to enumerate all extreme points. One can start from the MP with some bender-cut constraints associated with a subset of extreme points, and further solve a dual subproblem to add new Benders cuts to the MP. Various alternative BP strategies have been introduced (as reviewed in the paper^35^). We adopt the classic one to accelerate the computation as shown in Algorithm 1. As the DH can solve the problem within minutes, we utilize it to find the initial feasible solution when implementing the BP algorithm in our numerical test.
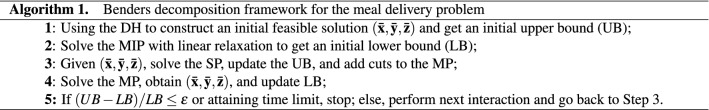


## Case study

We carry out a real-world case study of meal delivery by the IHCST in HK. First, we utilize real data to test the effectiveness of our modified 2E-VRP model, compared with the current manual approach. Second, we test how our robust model protects against uncertainty, i.e., how the delivery efficiency changes with the chosen level of robustness. Third, we illustrate and provide insight into how large is the price of robustness in our meal-delivery case. Finally, we discuss some perspectives and insights in terms of future demand growth. All the computational experiments are coded in Python programming language^36^ and the Gurobi 7.5.1 solver is used on a Windows 7 PC with an Intel Core i7 processor, 3.60 GHz CPU, and 16GB RAM.

An IHCST serves elderly clients living in neighboring communities in HK, with daily meal deliveries, including lunch and dinner. Some clients ask for both lunch and dinner and some ask for either. We collected related data and information (in 2017–2018) from the following sources: (i) consultations with delivery center managers, (ii) field observations from regular delivery sessions that provide time spent on routes and meal deliveries, (iii) maps that cover the delivery routes, van/bus stops, and buildings of clients, and (iv) a database of the clients, their living units and regular delivery staff of the IHCST. Travel time and service time duration for the frail and ordinary elderly were measured and recorded by a team who followed the delivery workers and marked the routes. The task assignment and routing by the IHCST were generated manually largely based on workers’ experience. We observed that the nearby buildings were served by workers directly on foot, while buildings farther away were reached by workers who first take via a delivery van and then on foot from the stops to their destinations. Compared with the manual approach in practice, we utilize the available data and measurements to test the effectiveness of our modified 2E-VRP models (i.e., the DM and RO). To ensure a fair comparison, we simulate with 5,000 replications that ordinary and frail elderly randomly realize to take their extreme values in terms of service time, and compare the average realized working time spent by workers under different approaches. Except for the uncertain service time, the daily elements of planning (e.g., the traveling time for each link and aggregate demand) are relatively stable in a short time. Hence, we focus on a typical day’s planning in December 2017 without losing generality. In total, the IHCST serves around 70 elderly clients spreading across 17 different buildings. Following the practitioners, the demand splitting parameter $\alpha$ is set as 0.5 and the additional penalty time is 1 min. In terms of the uncertain service time, we obtain the average service time for the ordinary and frail elderly (i.e., ${\bar{\mu }}_l^o$ and ${\bar{\mu }}_l^f$) based on the historical observations. As the service time is with highly unstable perturbations, we consider two sets of radius parameters for the test: a mild perturbation with ${\hat{\mu }}_o=80$ and ${\hat{\mu }}_f=160$ and a more severe perturbation with ${\hat{\mu }}_o=120$ and ${\hat{\mu }}_f=200$ (expressed in seconds). These perturbations were discussed and estimated together with the center’s staff and were taken as base cases. Take the first set of parameters as an example, we use a perturbation set of $[ {\bar{\mu }}_l^o - 80, {\bar{\mu }}_l^o + 80 ]$ for ordinary elderly and $[ {\bar{\mu }}_l^f - 160, {\bar{\mu }}_l^f + 160]$ for frail elderly.

**Table 4:** Average realized value with perturbation set $({\hat{\mu }}_o=80, {\hat{\mu }}_f=160)$.

**Session**	Realization	Realized value (min)			Percentage change (%)		
	$\%^o$, $\%^f$	Manual	DM	RO	$Gap_{M,DM}$ (%)	$Gap_{M, RO}$ (%)	$Gap_{DM, RO}$ (%)
Lunch	0.0, 0.0	50.2	47.9	47.9	−4.6	−4.6	0.0
	0.2, 0.2	54.2	52.2	52.7	−3.7	−2.8	1.0
	0.4, 0.4	57.7	55.4	52.6	−4.0	−8.8	−5.1
	0.8, 0.8	63.3	60.0	55.8	−5.2	−11.8	−7.0
	0.4, 0.8	61.6	58.3	59.7	−5.4	−3.1	2.4
	1.0, 1.0	66.2	61.3	57.9	−7.4	−12.5	−5.5
Dinner	0.0, 0.0	46.8	45.3	45.3	−3.2	−3.2	0.0
	0.2, 0.2	49.9	49.2	49.9	−1.4	0.0	1.4
	0.4, 0.4	54.1	52.3	52.5	−3.3	−3.0	0.4
	0.8, 0.8	61.3	57.2	56.9	−6.7	−7.2	−0.5
	0.4, 0.8	59.2	55.3	54.7	−6.6	−7.6	−1.1
	1.0, 1.0	65.5	59.5	58.1	−9.2	−11.3	−2.4
AVG		57.5	54.5	53.7	−5.1	−6.3	−1.4

### Comparison of results

**Figure 2:**
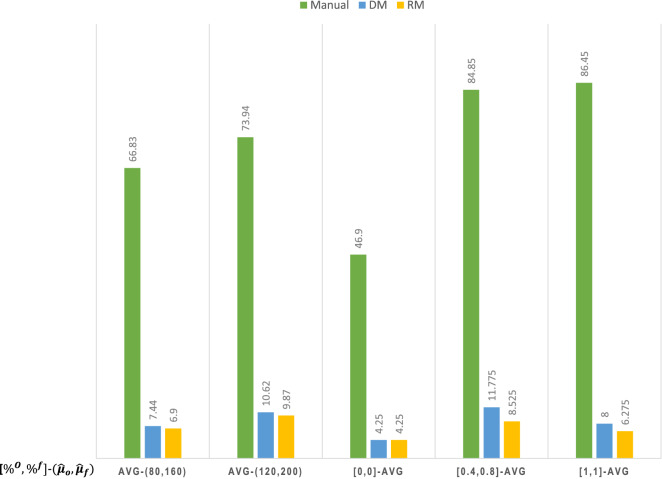
Variance of realized working time spent among delivery workers.

The results associated with two perturbation sets are shown in Table 4 (with ${\hat{\mu }}_o=80$ and ${\hat{\mu }}_f=160$) and Table 5 (with ${\hat{\mu }}_o=120$ and ${\hat{\mu }}_f=200$). The first column distinguishes between two delivery sessions (lunch and dinner). The second column shows the percentage of ordinary and frail elderly (i.e., $\%^o$ and $\%^f$) taking their extreme values in the simulation. We have $\%^o=\frac{\Gamma _o}{|L^o|}$ and $\%^f=\frac{\Gamma _f}{|L^f|}$ to represent the percentages with $\%^o, \%^f \in [0,1]$ . The next three columns show the realized objective values averaged over 5000 simulation runs of three approaches (i.e., manual approach, our DM, and RO), followed by three columns showing percentage change (i.e., $Gap^{M, DM}$, $Gap^{M, RO}$ and $Gap^{DM, RO}$) between the three approaches with $``M''$ denoting the manual approach. The manual approach is adopted by IHCSTs to generate task assignments and routing solutions based on experience. The bottom row shows grand averages across all shown realizations. As shown in Table 4 and 5, the average realized time spent by workers is about 3.0–4.5 min less for the DM and RO compared to the manual solution, with 4.7–7.0% improvement. Specifically, the improvement can be as large as 14.1% (for the case of $\%^o, \%^f=1.0$). When $\%^o, \%^f=0.0$ (i.e., no one takes the extreme value), the RO is equivalent to the DM, with a 4.6% improvement compared with the manual approach. We first conclude that our modified 2E-VRP model is effective in modeling meal delivery for the elderly in HK. As expected, the benefit of the RO becomes more evident when a higher percentage of clients take their extreme values, showing larger improvements over the DM. The DM outperforms the RO when the proportion of extreme values is small. This illustrates the price of robustness (to be discussed below). When the service time is subject to more severe perturbations (as shown in Table 5), the improvement of the RO is more significant, with an average of 7% improvement compared with the current manual approach. The improved efficiency is mainly attributable to our practical model capturing important real-world attributes. In practice, the written working time limit on each delivery session for each worker is an hour, corresponding to the length of the proper time window for customers to have meals. As shown in Tables 4 and 5, the realized time of the manual approach is very possible to be larger than 60 mins, especially when the service time is subject to a more severe perturbation. This means that some workers are overloaded and may feel an unfair workload, further leading to a high turnover rate and exacerbating the staff shortage issue. On the contrary, the realized time of our RO model is within 60 mins for most of the scenarios. Regarding workload fairness, we also calculate the variance of realized time spent among the delivery workers, as shown in Fig. 2. We observe that by fixing different parameters and taking an average of the variance of the realized time among workers, the variance value through the manual approach can be reduced significantly by introducing our models, especially the RO model. This means that the min-max objective format in our proposed models can well address the fairness issue regarding the workload of the delivery workers. Note that we also tested more instances on other various days and observed similar results as in Tables 4 and 5 and Fig. 2. Therefore, we further confirm the effectiveness of the RO model in protecting against uncertain service time, with a balance between delivery efficiency and workload fairness.

**Table 5:** Average realized value with perturbation set $({\hat{\mu }}_o=120, {\hat{\mu }}_f=200)$.

**Session**	Realization	Realized value (min)			Percentage change (%)		
	$\%^o$, $\%^f$	Manual	DM	RO	$Gap_{M,DM}$ (%)	$Gap_{M, RO}$ (%)	$Gap_{DM, RO}$ (%)
Lunch	0.0, 0.0	50.2	47.9	47.9	−4.6	−4.6	0.0
	0.2, 0.2	55.9	54.0	54.6	−3.4	−2.3	1.1
	0.4, 0.4	60.5	58.3	54.7	−3.6	−9.6	−6.2
	0.8, 0.8	68.0	64.7	58.5	−4.9	−14.0	−9.6
	0.4, 0.8	65.4	61.9	61.8	−5.4	−5.5	−0.2
	1.0, 1.0	71.5	66.6	61.4	−6.9	−14.1	−7.8
Dinner	0.0, 0.0	46.8	45.3	45.3	−3.2	−3.2	0.0
	0.2, 0.2	51.0	50.9	51.3	−0.2	0.6	0.8
	0.4, 0.4	56.6	55.2	55.6	−2.5	−1.8	0.7
	0.8, 0.8	65.9	61.8	60.2	−6.2	−8.6	−2.6
	0.4, 0.8	62.9	58.7	57.9	−6.7	−7.9	−1.4
	1.0, 1.0	71.5	64.8	62.2	−9.4	−13.0	−4.0
AVG		60.5	57.5	56.0	−4.7	−7.0	−2.4

### Price of robustness

Given the effectiveness of introduced robustness in our modified 2E-VRP model, we further analyze the price of robustness in terms of objective value, i.e., the reduction in the objective value from its nominal optimal value to its robust optimal value. As the levels of $\Gamma ^k_o$ and $\Gamma _f^k$ increase, the solution becomes more conservative and more robust against uncertain service times. Table 6 shows the price of robustness at different levels of chosen conservatism (as shown in the column “Robustness$''$), where the $``Obj''$ columns refer to the objective value of the RO. Note that this objective value is the worst-case value, different from the average “realized value$''$ in Tables 4 and 5. As shown in Table 6, the price of robustness ranges from 8.4 to 16.9 min. The benefit of using the RO as opposed to the DM increases with the perturbation scale and with more extreme realizations. This is particularly visible in the last column of Tables 4 and 5. Table 6 demonstrates that the price of robustness does not increase further after a certain level of robustness. Hence, whether we choose to be robust against 40% or against 80% of clients taking their extreme values, the worst-case objective value will be the same, although their realized values may differ. This provides us an insight that the center managers can be much more conservative without paying a high price of robustness when implementing the RO model.

The results inform practitioners to be risk-averse toward uncertain service times and uneven workloads. The robustness parameters $\Gamma _o$ and $\Gamma _f$ control the degree of conservatism that the user wants to exert. In practice, as we don’t know the exact magnitude of the permutation set in advance, we need to consider some practical methods to determine the parameters, although the price of robustness is not high. Some standard procedures could be followed to choose a proper level of conservatism against uncertainty^37^. One can consider a validation method by introducing a randomly generated validation set of historical observations to help determine these parameters. For example, following the standard procedure, we can divide the historical data into a training set and a validation set with a ratio of 0.8:0.2.

**Table 6:** Price of robustness.

	Robustness	$({\hat{\mu }}_o=80, {\hat{\mu }}_f=160)$		$({\hat{\mu }}_o=120, {\hat{\mu }}_f=200)$	
	$\%^o$, $\%^f$	*Obj*	*Price*	*Obj*	*Price*
Lunch	0.0, 0.0	47.9	0.0	47.9	0.0
	0.2, 0.2	57.1	9.2	60.1	12.2
	0.4, 0.4	60.2	12.3	64.1	16.2
	0.8, 0.8	60.2	12.3	64.8	16.9
	0.4, 0.8	60.2	12.3	64.8	16.9
	1.0, 1.0	60.2	12.3	64.8	16.9
Dinner	0.0, 0.0	45.3	0.0	45.3	0.0
	0.2, 0.2	53.7	8.4	57.6	12.3
	0.4, 0.4	58.1	12.8	62.2	16.9
	0.8, 0.8	58.1	12.8	62.2	16.9
	0.4, 0.8	58.1	12.8	62.2	16.9
	1.0, 1.0	58.1	12.8	62.2	16.9

### Computation efficiency

In terms of computation efficiency, we report and compare the objective values obtained by our two decomposition-based approaches (i.e., DH and BD) with a CPU time limit set as 15 min and directly by the solver (i.e., Gurobi) with a CPU time limit set as 30 and 60 min in Table 7. We observe that our approaches (DH15 and BD15 in Table 7) can effectively accelerate the computation, obtaining better results within the less CPU time limit compared with being directly solved by Gurobi (i.e., Solver30). The practical planning of routing and assignment is operated daily, and thus the computation time before daily delivery should be controlled within a short time. Notably, the decomposition-based heuristic (i.e., DH) performs so efficiently, obtaining high-quality solutions within just a few minutes.

**Table 7:** Objective value of RO model through different approaches.

	Robustness	$({\hat{\mu }}_o=120, {\hat{\mu }}_f=200)$			
	$\%^o$, $\%^f$	Solver30	Solver60	DH15	BD15
Lunch	0.0, 0.0	47.9	47.9$^*$	47.9	47.9
	0.2, 0.2	65.8	60.1	60.1	60.1
	0.4, 0.4	74.6	64.1	64.1	64.1
	0.8, 0.8	79.5	64.8	66.3	64.8
	1.0, 1.0	79.5	64.8	66.3	64.8
Dinner	0.0, 0.0	45.3$^*$	45.3$^*$	45.9	45.3
	0.2, 0.2	60.4	57.6	57.6	57.6
	0.4, 0.4	63.1	63.1	62.2	62.2
	0.8, 0.8	63.1	63.1	62.2	62.2
	1.0, 1.0	63.1	63.1	62.2	62.2

$*$ means that the problem is optimally solved within the specified time limit.

**Figure 3:**
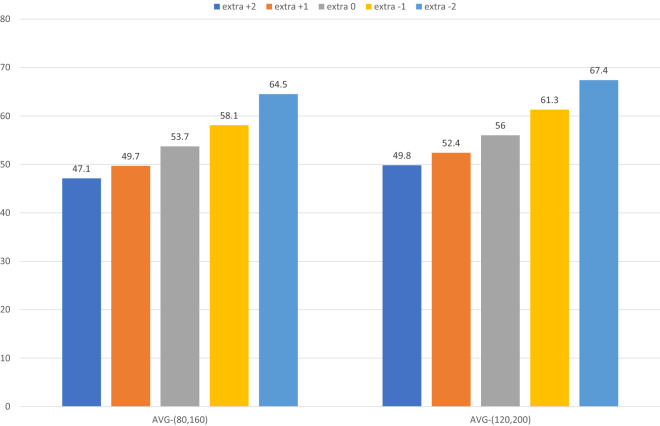
Realized objective value for 0, ±1, and ±2 workers.

### Sensitivity analysis

We also conduct sensitivity analysis concerning having $\pm$1 and $\pm$2 workers. Figure 3 shows the realized objective value for 0, 1, −1, 2, and −2 extra workers, across different perturbation levels. With the current number of workers, the objective value is always under 60 min. Note that the current number of workers is determined by $\sum \limits _{i \in B}\lceil |L_i|/(0.5\times C)\rceil = 19$ (that has been applied in practice). With one extra worker, the realized objective value remains under 55 min, and with one less worker, it can attain 61.3 min on average and even attain 67.4 min for the extra −2 case. This information can help the practitioner understand the effect of adding or removing workers to the workforce, given certain assumptions about perturbations and chosen levels of conservatism.

### Demand growth

Demand for home care services is expected to grow, and we assess the effect on our model’s objective value and computation efficiency. We have a test session with a demand of 98 clients spread across 20 buildings. In our test, we observe that the RO directly solved by Gurobi cannot achieve the optimal (or even obtain very bad solutions) within the 1-h time limit for all of the instances. With our DH approach, we can find better solutions for the RO within several minutes. Table 8 shows average realized values after 5000 simulations with different levels of conservatism under the perturbation set [80, 160]. We observe that the RO (solved by the DH) can achieve more benefit than the DM with on average 5.6% improvement in realized value when the demand becomes higher in comparison with small-scale problems (as shown in Tables 4 and 5). We also observe that the average realized makespan is much shorter than 60 mins, meaning that the human resources can be better utilized under scientific approaches and the positive effect of the modified 2E-VRP modeling is amplified with growing demand. This provides insights into long-term human resource planning for decision-makers. That is, the parameter $\alpha$ in staffing formula $\sum \limits _{i \in B}\lceil |L_i|/(\alpha \times C)\rceil$ can be modified to a larger value (with $0.5\le \alpha \le 1$) when the demand is growing. The resource aggregation effect under scientific approaches can partially offset challenges faced in demand growth for meal delivery.

**Table 8:** Average realized values under high demand and perturbation set $({\hat{\mu }}_o=80, {\hat{\mu }}_f=160)$.

Realization	Realized value		Percentage
$\%^o$, $\%^f$	DM	RO	$Gap_{DM, RO}$ (%)
0.0, 0.0	37.5	37.5	0.0
0.2, 0.2	39.6	38.9	−1.6
0.4, 0.4	41.5	39.8	−4.1
0.8, 0.8	45.6	41.8	−8.3
0.4, 0.8	44.9	41.0	−8.7
1.0, 1.0	47.9	42.6	−11.0
AVG	42.8	40.3	−5.6

## Concluding remarks

The current context provides a case study of robust optimization applied to a practical problem of home care services, which is uniquely modeled as a modified 2-echelon vehicle routing problem (2E-VRP). We provide a scientific approach for decision-makers to determine the appropriate routing and workload allocation to improve delivery efficiency, with different levels of conservatism. Specifically, our model and analysis have incorporated important practical aspects, such as fairness among workers, “continuity of care”, and features of buildings. Notably, we differentiate between the frail and ordinary elderly in terms of uncertain service time, which is mainly addressed in our focal robust 2E-VRP model. We also provide practical decomposition-based approaches to accelerate the computation of the robust 2E-VRP model, which is transformed into a mixed integer program. Through numerical analysis, we verify the value and effectiveness of our proposed 2E-VRP models in the meal-delivery context, compared with the current practice. The structures of our models can also be applied to general community-based home care services (such as housekeeping and medical examination) in any densely populated and aging city like Hong Kong. Our case study results show that robust solutions can protect against service time variations and realize better performance while incurring a small additional cost over deterministic optimal solutions. As demonstrated, the benefit of using robust optimization also increases as larger perturbations apply. With our scientific approach against uncertainty, the improvement in delivery efficiency can further reduce the huge burden on the already cash-strapped delivery team and better coordinate with resources in integrated care services. The results and analysis also shed light on risk management (e.g., determining the level of conservatism) and long-term human resource planning.

One of the future directions is to integrate multiple home care services into a single optimization effort. It would be advantageous if different services, such as housekeeping, medical examination, and meal delivery, can be coordinated and optimized simultaneously^3^. In this case, a van assisting workers in improving on-road transportation between different buildings (instead of walking) can be considered to improve the efficiency of the delivery system. The second direction is to investigate delivery economies where vehicles and staff are tracked in real-time and planning decision epochs are much more dynamic and granular. Considering a dynamically updated delivery system may better protect against real-time variable service times. As real-time decision-making largely depends on the internet of things (e.g., sensors) to obtain real-time information, designing a system that is remotely programmable to implement optimization algorithms within the sensor-based network can also be an interesting direction^38^. Another practical direction is to incorporate the elements of ride-hailing service^39^ into the meal-delivery system. A discussion on efficiency and fairness under such a new context would be promising.

## Data Availability

The onsite delivery data and simulation data that support the findings of this study are available at reasonable request from the corresponding author. The data are not publicly available because they contain information that could compromise the privacy of research participants. Due to the nature of privacy issues, supporting data about the detailed information of buildings and clients in this study are not available.
